# The health perception of urban green spaces and its emotional impact on young adults: an empirical study from three cities in China

**DOI:** 10.3389/fpubh.2023.1232216

**Published:** 2023-11-02

**Authors:** Jinsong Wang, Nan Liu, Jiaying Zou, Yanlong Guo, Hong Chen

**Affiliations:** ^1^Department of Design, College of Art, Anhui University, Hefei, Anhui, China; ^2^Department of Design, College of Art, Jiangxi University of Finance and Economics, Nanchang, Jiangxi, China

**Keywords:** urban green space, emotional health, green space perception, influencing, young people

## Abstract

**Introduction:**

Exposure to green space can bring many benefits to physical and mental health, but in China, the attractiveness of green space to youth groups seems to be not significant. The question of how to encourage young people to go out of the house to better perceive green space, enjoy nature, and promote physical and mental health is on our minds.

**Methods:**

This study combines young people’s green space perception, green space use, and purpose of visit to explore its impact on the emotional health of youth groups, combined with the PANAS psychological data scale, an online questionnaire survey of 426 residents (18–35  years old) in three Chinese cities, and was used to construct a multiple regression model and AMOS structural equations.

**Results and discussion:**

The results of the study showed that, firstly, environmental attractiveness, environmental odor, and number of facilities were the key factors influencing adolescents’ landscape perception evaluation, while activity space, environmental odor, and environmental attractiveness had a greater impact on adolescents’ emotional well-being. Second, among adolescents’ visit purposes, socializing and fitness were more likely to help them generate positive emotions while resting and viewing activities were effective in helping them alleviate negative emotions. In addition, in terms of usage, residents who took public transportation as well as those who arrived on foot were the most emotionally healthy. The findings of this paper provide insights for public policymakers, urban planners, and landscape architects to better encourage youth participation in green spaces when they are installed.

## 1. Introduction

With increased urbanization and growing life stress, people’s health problems, especially mental health problems, are becoming more and more serious (1–3). In previous research surveys, it is even found that the proportion of young people suffering from mental illness is quite large, and as of 2019, there are nearly 1 billion people worldwide suffering from mental disorders, including stress, anxiety, mental fatigue, and depression, including 13% of young people, and in the first year of experiencing the pandemic, depression, and anxiety disorders increased by 25% in just the first year of the pandemic (4). In the 2022 China National Mental Health Survey, the risk detection rate was 10.6% for depression and 15.8% for anxiety. Among the adult population, youth are a high-risk group for depression, with a significantly higher rate of 24.1% in the 18–24 age group and 12.3% in the 25–34 age group (5). Mental health problems in youth groups are a serious worldwide problem that, if not addressed on time, will continue to affect the health of the nation’s population (6), making them susceptible to mental illnesses and concomitant conditions, including behavioral and emotional problems (7).

In addition to pharmacological treatment and control, it is widely believed that mental health and emotional well-being can be restored in the natural environment (8–11). Therefore, given the global mental health burden, of which mental, neurological, and substance use disorders account for 13% of the total disease burden (12) and the importance of addressing the emotional health of youth (13), we must improve our understanding of the relationship between the natural environment and the emotional health of youth. In conjunction with the Outline of China’s Medium-and Long-term Youth Development Plan (2016–2025), we focused our study on urban youth aged 18 to 35 years (14) on exploring the perceived benefits and emotional impact of using green space on youth in three cities, Hefei, Guangzhou, and Xi’an, China, based on the Chinese Urban Green Space Classification Standard (CJJ/T 85–2002), we have studied four categories of urban parks: municipal parks, district parks, community parks, and street-side strip parks. The PANAS scale was used to analyze the emotional health of urban green space and youth groups to explore the significant effect of urban green space on improving emotional health; after that, we will further construct structural equation models of the effects of green space perception, the purpose of visit, and green space use on emotional health, and further test the hypotheses through experiments. It is hoped that this study will provide a reference for improving the urban green space environment, provide constructive suggestions for green space planning, increase the enthusiasm of young urban residents to use parks, promote the participation of the youth population in green space activities, and improve physical, mental, and emotional health.

### 1.1. Literature review

#### 1.1.1. Emotional well-being

Emotional Well-Being (EWB) as a scientific and public health concept is not synonymous with the absence of disease, nor is it synonymous with mental health. EWB, an umbrella label for several related psychometrically defined concepts, includes positive mental health, healthy quality of life, and subjective well-being. Positive emotions and moods (e.g., happiness), the relative absence of negative emotions, moods and states (e.g., stress, sadness, loneliness), life satisfaction, sense of meaning and purpose, quality of life, and satisfaction with other life domains (e.g., job satisfaction, relationship satisfaction) are all important indicators of emotional health (15). The distinction between positive affect (PA) and negative affect (NA) encompassed by emotional health is considered a key factor in explaining the high correlation between measures of anxiety and depression and is an important aspect of mental health (16). Countries around the world are also gradually recognizing the importance of emotional health, and as early as 2011, the National Prevention, Health Promotion, and Public Health Council in the United States listed mental and emotional health as one of the seven priority areas of the National Prevention Strategy to increase public attention to emotional health (13); New Zealand in 2013 identified emotional health as an important observation for reducing social and psychological morbidity in adolescence (15). Observing nature can positively impact people’s emotions, according to the field of psychological and evolutionary theory (17); exposure to nature can also improve how people perceive nature and lessen unpleasant emotions like anger, fear, and confusion (18).

#### 1.1.2. Emotional well-being and green space

This is well demonstrated by the Attention Recovery Theory (ART) proposed by Kaplans and the Stress Reduction Theory (SRT) on restorative environments proposed by Ulrich, whom both contend that natural settings can serve as a vehicle for restorative settings (19), i.e., they can elicit easy attention and permit the recuperation of worn-out voluntary attention and mental weariness. When observing their surroundings, a person first experiences an emotional reaction (such as likes or dislikes), which then triggers cognition (such as an appraisal of the scene to determine if it is helpful or harmful) and emotional arousal (such as negative or positive feelings) (20). This then affects behavior and physiological arousal (such as relaxation) (21). Many studies on the positive benefits of green space for mental health have emerged based on the classic theories of ART and SRT, and both fully substantiate this idea (22–26).

The degree of emotional restorative qualities of the interior elements of green spaces has been studied experimentally by students at the University of Pennsylvania through a simulation experiment in which student subjects were asked to listen to natural sounds, artificial noise, and controlled sounds after a memory depletion task, and the results showed that natural sounds did indeed lead to better restoration of attention (27); a Mexican study on the biophilic and restorative qualities of indoor and outdoor spaces also showed that environmental quality was associated with a decrease in negative emotions and stress perceptions and an increase in positive emotional states (28); and British scholars found that sounds in the forest stimulated the most blissful responses by bringing subjects into the forest to experience it firsthand (29). There is also an assessment of the differences in the strength of different types of green spaces on the restorative emotional health of groups of people, and then in a Swedish experimental study comparing the extent to which visual stimuli, olfactory stimuli, and auditory stimuli in dense urban areas, parks, and forests affected physiological stress recovery, the results showed that high pleasantness of the environment was associated with a low physiological stress response to olfaction and audition and that olfactory stimuli may be more helpful than visual stimuli in reducing stress (30); a study in China compared the strength of grass, square, forest and lakeside on people’s restorative level, and found that lakeside and forest showed the strongest restorative properties in terms of reducing negative emotions and increasing positive emotions (31). There are also studies on the level of emotional health of a certain group of people in the green, Jeayoung et al. studied the role of auditory and visual elements in campus green spaces on the emotional health of college students, showing that high biodiversity and natural sounds are beneficial to the recovery of mental and emotional health of the college population (32); Chinese scholars collected data from college campuses in two different provinces, proving that during the pandemic period, campus Chinese scholars collected data from university campuses in two different provinces, demonstrating the positive impact of natural elements such as plants, lawns, and bodies of water in green spaces on the emotional health of university students during the pandemic (33). In Barcelona, a hands-on investigation of the total green space environment in which elementary school students in grades 2–4 live and learn demonstrated that the greenness of the space was associated with children’s emotional well-being (34); and in New Zealand, the effects of blue, green, and biodiversity on adolescents’ mental and emotional well-being were assessed in a community setting, and the results showed a positive correlation between the two (35). Through combing through the literature, we found that the research population focuses on children (36), college student groups (37), and older adults (38), while little research has been done on young urban residents aged 18–35 in China, which we would like to know. Moreover, the current research has explored the restorative effects of natural elements in blue space and green space on emotional health (39), while ignoring the connection between other objective conditions and the subject’s own use of the green space and emotional health.

#### 1.1.3. Perceived benefits, green space use and purpose of visit

Green space perception refers to the public’s perception of the internal quality aspects of green space, and related studies have shown that this perception is associated with the level of well-being, meaning that when people perceive more green space, they will have a better level of mental and emotional well-being, and at this point they also reap more green perceived benefits (40–42); the purpose of the visit, i.e., the motivation for visiting the green space, includes exercise, socialization, relaxation, and other aspects, different types of motivation are associated with the perceived outcomes of nature visits, with relevant studies showing that 5 min of exercise in a natural environment can improve mood and self-esteem (43), stress reduction motivation and increased emotional well-being are the strongest associations (44); green space use includes aspects such as frequency of visits to green spaces, accessibility, and duration of visits, which have been demonstrated to be associated (45–47).

#### 1.1.4. The PANAS scale

The Positive and Negative Affect Scale (PANAS) is a simple and easy-to-administer version of the most widely and commonly used scale for assessing positive and negative affect, originally developed and designed in North America by Watson et al. in 1988, and has since been validated in many cities and shown excellent psychometric properties in several studies (48), such as in Santiago, Chile, where researchers used the PANAS scale to measure the impact of small-scale green infrastructure on people’s emotional well-being (49); American scholars used the PANAS scale to measure the impact of the length of time spent in nature on the emotional well-being of schoolchildren (50); and Chinese scholars used the PANAS scale to measure differences in the restorative effects on young people of four different green spaces (31). The scale is designed to measure positive and negative emotions in a person’s current state. The final score is derived from the sum of 10 items for each of the positive and negative describing mood, stress, and spirituality, and they may reflect to some extent changes in the subject’s mood (48).

### 1.2. Research aims and objectives

Inspired by these previous studies, the present study aims to explore the significant effects of urban green spaces on improving emotional well-being through the PANAS scale. Further, we planned to develop a structural equation model to analyze the effects of green space perception, purpose of visit, and green space use on emotional well-being using them as variables. In view of this, we constructed a research framework to link green space perception, purpose of visit, and green space usage to emotional well-being, and plan to test the hypotheses through a field study. This study can enrich the research on greenfield health in China and make more scholars pay attention to the mental health and emotional health problems of youth groups, which will have a positive contribution to the improvement of the emotional health and mental health of youth groups. We expect that this study will provide a useful reference for improving the urban green space environment, provide constructive advice for green space planning, increase the motivation of young urban residents to use parks, exposure to green space, and promote youth participation in green space activities to improve emotional health. More specifically, we attempted to elucidate the health perceptions of urban green spaces and their relationship to the emotional health of the youth population through a survey study from the following questions:

1. What aspects of the perception of the health of urban green areas are more important to young people?
2. Which green space health components have an impact on the emotional health of young people?
3. What are the effects of green space use behavior characteristics on the emotional health of young people?
4. What is the relationship between the perceived benefit of green space, visit purpose, and emotional health?

## 2. Materials and methods

### 2.1. Study site

In this paper, convenient sampling survey was conducted by distributing an online questionnaire to young people in three cities (Supplementary Figure S1): Hefei, Guangzhou, and Xi’an, located in the Yangtze River Delta, Pearl River Delta, and Guanzhong Plain, respectively, representing cities in the central, southern, and northern regions of China. Hefei abbreviated as “Lu” or “He,” was known in ancient times as Luzhou. Hefei is a prefecture-level city, and also the capital of Anhui Province. By 2021, Hefei has a registered population of 7,926,700 and a permanent resident population of 9,465 million. The urban green coverage area is 25,195 hectares, and the green coverage rate reaches 44.18% (51). The total area of Guangzhou is 7434.40 square kilometers, with a registered population of 10.1153 million and a permanent resident population of 18.810,600. The green development road of “greening and returning people and scenery to people” is accelerating the construction of the core garden area in Guangzhou. A total of 3,800 kilometers of greenways, an increase of 6.7% over the previous year, 6.2 kilometers of new cloud roads, 43 pocket parks, 43 kilometers of greenways, 400 kilometers of blue roads, 38.26% of green coverage in built-up areas, urban green coverage area of 59,558 hectares, ranking in the forefront of the country’s first-tier cities, the popularity of “Flower city” in Guangzhou has increased significantly (52). Xi ‘an is the capital of Shaanxi Province and an important educational, industrial and scientific base in northwest China. Xi ‘an is a city with a population of 13.1630 million and rich in natural resources. By the end of 2021, the urban green space coverage rate of Xi‘An will reach 42.72%, with 46,466 hectares of urban green coverage and 149 urban parks (53).

As shown in Supplementary Table S1, the cities of Hefei, Guangzhou, and Xi’an, which are typical representatives of Chinese cities with similar green coverage and a rich variety of green spaces, were selected as the sites for this study.

### 2.2. Procedures and participants

As of June 2022, China has 1.051 billion Internet users, with an Internet penetration rate of 74.4%, of which about 50% are 10–39 years old (54). For the survey of young people, online surveys may be more likely to elicit responses from young people. Therefore, we adopted www.wjx.cn, one of the most famous online data collection platforms in China, for questionnaire distribution and management. The questionnaire was sent to potential participants for invitation via social media platforms and email between April and June 2022, and participants were informed that the study required eligibility between the ages of 18–35, while at the same time must have lived in the city for more than 6 months, with a link to the invitation or a QR code (55).

The questionnaire detailed the research purpose, data subjects, data use, and information anonymity, and participants filled out and submitted the questionnaire with informed consent. Participants are also given security assurances that the questionnaire information will be kept confidential and used for non-commercial purposes, an assurance that helps build trust and encourages participants to answer questions honestly. In this study, it is ensured that all processes carried out in the study comply with ethical principles and the legal requirements of the People’s Republic of China. After 2 months, we collected 502 online questionnaires, of which 76 were invalid due to the selection of contradictory options and short response time, and the remaining 426 were valid, with an overall response rate of 84.8% (invalid response rate of 15.2%).

### 2.3. Questionnaire content

Through research understanding of the literature, a web-based questionnaire was designed to collect data containing 38 questions divided into five modules (1): demographic information (2); characteristics of green space use (3); purposes of visiting green spaces (4); attitudes about the advantages of green spaces (5); levels of emotional well-being investigated through the PANAS scale. In the first part, the aim was to collect demographic data such as gender, age, and city. The next section was to understand the characteristics of people’s use of green spaces, including frequency of visits, time spent in activities, and time spent. The third part is to investigate the purpose of people’s use of green spaces and whether there is an association between different purposes of use and emotional well-being. The fourth part was to understand people’s perceptions of the benefits of green space, with the aim of following up on whether the level of perceptions had an impact on emotional health, and the fifth part was to investigate people’s emotional health using the PANAS scale.

With demographic information and use characteristics as control variables, the purpose of use and green space perception as independent variables, and emotional health as dependent variables. The dependent variable is the response value of participants to their emotional changes after they pass the independent variable of green space benefit cognition and their use behavior. Control variables are sometimes referred to as extra correlated variables, which refer to possible influences on experimental outcomes besides independent and dependent variables. The time to complete the questionnaire was approximately 5–15 min per respondent.

#### 2.3.1. Dependent variables

In the dependent variable section, the PANAS psychological data scale was used. The PANAS consists of 20 emotion words, which were streamlined to 10 emotion words in the questionnaire and consisted of 5 positive emotion items and five negative emotion items. Higher values of the items indicate a higher level of emotion in the subject; for example, a higher positive emotion score indicates a higher level of positive emotion in youths, while a higher negative emotion score also reflects a high level of negative emotion in youths (48).

#### 2.3.2. Independent variables

The section on green space perception focuses on the perceived effect of green space on emotional health (Supplementary Table S2). The effect of green space on emotional well-being in the health-promoting green space section was broken down into perceptions of green space attributes (two items), landscape features (five items), and facility management (three items). All three sections were based on previous research for greenspace attributes; participants were asked about greenspace activity spaces (activity plazas and open spaces), pathway systems (road planning, road paving) (56), and whether they had an impact on mood. Landscape attributes were assessed by asking whether the botanical landscape (color scheme of plants, number of species, ornamentation) (42), environmental smells (plant odors and soil aromas), and environmental sounds (animal calls, water flow, music) (57–59), environmental attractiveness (environmental tidiness) (60), and animal groups (wildlife in green spaces) affected mood (35). In terms of facilities, participants were asked whether the management system of the green space (environmental safety, activity arrangements) (61), the number of resting (chairs and seating), and recreational facilities (running track, sports facilities, interactive facilities) and the quality of the facilities (whether the facilities were intact and stable) affected the respondents’ mood (56, 62).

Responses to the question on the positive impact of green space on mental health were on a five-point Likert scale (1 = strongly disagree, 5 = strongly agree) with increasing levels of perception from 1 to 5. Characteristics of green space use included frequency of visits, time spent on visits and activities, type of activity, mode of transportation, and time spent. Motivations for youths’ visits included stress relief, physical activity, and social recreation. This part of the study examined the effect of post-use on changes in emotional well-being using the PANAS scale to understand respondents’ perceived levels of green space, characteristics of green space use, and the purpose of visits.

#### 2.3.3. Control variables

In the control variables section, six demographic characteristics, such as gender, age, income, city, education, and occupational status, were used as control variables by reading the previous results because the emotional health of the youth group is closely related to the sociological status of the individual. The analysis was conducted by using control and explanatory variables as linear regressions. The frequency of visits was also used as a control variable in the structural equation modeling analysis.

#### 2.3.4. Data processing

Descriptive statistics are used to analyze the distribution of a sample across demographic and behavioral variables. SPSS 24.0 statistical software was used for analysis. Multiple regression analysis and analysis of variance were used to determine the correlation between the social demographic characteristics and green perception of the respondents, the characteristics of using green space, the purpose of visiting green space, and emotional health, so as to test the moderating effect of these variables on the emotional health of young residents. SPSS 24.0 statistical software was used for analysis.

To explore the correlation between perceived benefit, interview purpose, and emotional health, first, the α coefficient test was used to evaluate the reliability of the scale. Then, factor analysis was used to test the applicability of the structural model, and statistical software AMOS 24.0 was used for statistical analysis. The structural model showed that RMSEA<0.08, CFI, IFI, NFI, and GFI > 0.85, indicating that the structure has a good fit (63, 64).

## 3. Results

### 3.1. Reliability and validity analysis

The Cronbach’s alpha coefficient for the questionnaire items was 0.751, and in theory, a value greater than 0.7 indicates that the questionnaire has a high degree of reliability (65). This indicates that the questionnaire has good reliability and high internal consistency. The validity of the questionnaire was also tested, the KMO must be greater than 0.7, and Bartlett’s sphericity must be less than 0.01 (66). Through data analysis, the KMO value was 0.847, and the result of Bartlett’s sphericity test was 0.000. It shows that the structure of the test is very valid, and the correlation between variables is found, which can be used for factor analysis.

### 3.2. Descriptive statistics

According to the statistics in Supplementary Table S3, out of 426 youth groups, 158 males, or 37% of the respondents, and 268 females, or 63% of the respondents, had a larger proportion of female respondents, probably because females were more willing to participate in the questionnaire than males (67). In terms of age structure distribution, there were more people aged 18–25 years old, with 254 people accounting for 60%, while there were fewer people aged 26–30 years old and 31–35 years old, accounting for a total of 40%.

The demographics section also includes frequency of visits, distance of visits, duration of visits, as well as specific questions related to green space use such as how they reached the green space, what kind of activities they came to primarily engage in, and how long they stayed in the green space.

### 3.3. Awareness of green space benefits

The type of activity 31.06% of the residents walk in green spaces, followed by resting and viewing in higher numbers, accounting for 20.85 and 20.76% of the total number of youth in this category, respectively, while the number of social and fitness youth is relatively low, accounting for 15.78 and 11.54% of the total number of youth, respectively, Supplementary Table S4 shows that, according to the results of the data analysis, the surveyed youth rated the effect of green spaces and emotional health higher with the mean value above 3.5. Environmental attractiveness, number of environmental odors and facilities, as well as the quality of facilities, management system, and plant landscape were the key factors influencing youth scores with mean values above 3.6, while the low scores for species diversity of fauna in green spaces indicate that fauna in green spaces has a relatively small impact on the perceived effect on residents’ health.

Positive and negative emotions of youths were counted and discussed separately through the PANAS psychological evaluation scale. By correlating the scores of green space perception and positive and negative mood measures, we can find that activity space showed a positive correlation with youths’ positive mood, and activity space, as well as environmental attractiveness, showed a significant negative correlation with environmental odor and negative correlation, with environmental odor showing more significant.

### 3.4. Analysis of the use of characteristic factors

Multiple regression analysis was conducted with the frequency of visit, activity time, mode of transportation, and time spent as dependent variables, while three independent variables, namely demographic characteristics, the purpose of visit, and perceived emotional health benefits of urban green spaces, were included in the regression model (68, 69). The final results from Supplementary Table S5 reveal that gender, occupation, and demographic variables have a significant negative effect on visit frequency; environmental odor has a prominent positive effect on visit frequency in greenspace perception; occupation and income demographic variables contribute positively to the mode of transportation at the time of activity and to the youth population, and environmental odor has a positive effect on youths’ mode of transportation in their urban greenspace health perception.

According to the results in Supplementary Table S6, we can find that walking has the highest proportion, with 375 people, accounting for 31.10% of the total number of participants. The lowest proportion of leisure and viewing, at 251 and 250, accounted for 20.80 and 20.70% of the total population, respectively. The number of fitness participants was 139, or 11.5% of the total. Young people engaged in fitness activities had the highest positive emotion scores, while those who engaged in ornamental activities had the lowest negative emotion scores.

Differences between groups were analyzed using the Kruskal-Wallis H test, which uses ranks of continuous variables (70, 71). Kruskal-Wallis H-test analysis was conducted using the frequency of visitation, activity time, and duration of visitation as group variables and PANAS positive and negative mood scores of the interviewed youths as test variables and the results of the study are presented in the Supplementary Table S7, where the frequency of visitation, activity time, duration of visitation and negative mood were indicated as significant (*H* = 6.478, *p* = 0.039 < 0.05), (*H* = 14.654, *p* = 0.005 < 0.05), (*H* = 14.654, *p* = 0.005 < 0.05), (*H* = 14.654, *p* = 0.005 < 0.05), (*H* = 14.654, *p* = 0.005 < 0.05), (*H* = 14.654, *p* = 0.005 < 0.05).

According to Supplementary Table S8, a one-way study was conducted to investigate differences in negative and positive health scores in the population, with the dependent variables being positive and negative emotional health scores on the youth PANAS and the independent variables being the mode of transportation and time spent (72). In terms of positive mood, the data used in the study conformed to a normal distribution within each group, with a chi-square test for the mode of transportation (*F* = 2.1, *p* = 0.064 > 0.05), tested by ANOVA. In terms of negative mood, the chi-square test for both variables was a homogeneity test with non-uniformity of variance (transportation, *F* = 2. 53, *p* < 0.05; time spent, *F* = 3.262, *p* < 0.05), so the Welch test was performed, and the Welch tests for both transportation and time spent in the table were significant (*p* < 0.05), indicating that the differences in scores by transportation and by time spent were significant. Residents who took the bus and subway and walked were the healthiest emotionally, while those who spent 31 min to 1 h on the journey were the healthiest emotionally.

### 3.5. The relationship between perceived emotional health gains, visitation motivation, and emotional health in greenfield

This is shown in the following Supplementary Table S9. The alpha coefficients of the latent variables for each dimension ranged from 0.741–0.936, and the overall Cronbach’s alpha coefficient was 0.848, indicating that the internal consistency between the observed terms in the latent variables was good and the statistics were plausible for factor analysis (73, 74).

The relationship between the variables was further assessed using structural equation modeling, which is also more appropriate due to the large number of variables under study. In this chapter, the equation model was constructed with the emotional health benefits of green space (referred to as green space perception) and purpose of visitation as latent variables, visitation frequency, activity time, and resident demographic characteristics (gender, age, and income), and then positive and negative emotional health as latent dependent variables, each with multiple observed variables to measure. Among the green space perception factors, water landscape, flora, fauna, number of facilities, quality of facilities, activity space, pathway system, recreational attractiveness, odor environment, sound environment, and green space supervision and management were used as observed variables; among the visit purpose factors, relaxation, exercise, and social recreation were used as observed variables. In addition, the five items describing positive emotions in the PANAS scale were used as the observed variables for positive emotional health, and the five items describing negative emotions were used as the observed variables for negative emotions. The structural equation model diagram is shown in Supplementary Figure S2.

Based on the structural model to analyze and process the data (Supplementary Table S10), the model fit indicators at this time are *X*^2^/df = 2.629 < 3, GFI = 0.895, AGFI = 0.869, CFI = 0.939, NFI = 906, IFI = 0.939, RMSEA = 0.062, implying that the structured factor relationships and the accommodation between the data are good and the model is accepted. According to the results of structural equation modeling analysis, green space perception has a significant positive effect on positive emotion (*p* < 0.05) with an impact coefficient of 0.078; green space perception has a significant effect on negative emotion (*p* < 0.05) with an impact coefficient of-0.127; visit purpose has a significant negative effect on negative emotion (*p* < 0.05) with an impact coefficient of 0.195. The impact coefficient was 0.380, and the purpose of the visit had a positive effect on positive emotions (*p* < 0.05).

Gender, age, and income control variables were added to the structural equation model for reciprocal analysis, as shown in Supplementary Table S11, to investigate whether gender, age, and income differences affect the emotional health of the population. The collected data were first processed to consider 18 to 25 years of age as the transition to adulthood as the lower age group (75), 26 years old and above as the high age group, monthly income below 5,000 as a low-income group, and monthly income above 5,000 as a high-income group. In terms of gender, the effect of green space perception on positive emotion was greater for men; the effect of green space perception on negative emotion was greater for women; the effect of the purpose of visit on both positive and negative emotion was greater for men than for women; in terms of age, the effect of green space perception on positive and negative emotion was greater for the younger group. Moreover, the purpose of the visit had a greater impact on positive emotions in the lower age group and a greater impact on negative emotions in the higher age group; in terms of income, green space perception had a greater impact on positive and negative emotions in the lower income group; the purpose of the visit had a greater impact on positive emotion in the lower income group and a greater impact on negative emotion in the youth of higher income level. From the statistical results, among the men under 25 years old whose income is less than RMB 5,000, the perception of green space and visiting purpose have the most obvious influence on emotion.

The frequency of visits and activity duration were used as covariates in a structural equation model for a multigroup analysis to examine whether the frequency of green space visits and activity duration among youths affected the emotional well-being of residents (Supplementary Table S12). The collected data were first processed, with visits to green spaces less than twice per week classified as low frequency, visits three or more times per week classified as high frequency, activities of 30 min or less and 30 min to 1 h classified as low duration, and 1 h to 2 h, 2 h to 4 h, and 4 h or more classified as high duration.

## 4. Discussion

### 4.1. Perceptions of the benefits of green space

The study found that the young people’s perception of green space is correlated with their emotional health, consistent with some studies from previous studies (76), and it is worth noting that young people surveyed showed a high level of concern about the attractiveness of the environment, the smell of the environment, and the number of facilities. This suggests that when young people are in a bad mood, young people tend to associate the environment with the potential to enhance their emotional well-being, especially through pleasant smells and the convenience of facilities.

In addition, our study showed that specific types of activity Spaces within green zones significantly enhanced the positive emotional states of young participants. This result may be attributed to the coexistence of activity Spaces within these public areas, which act as social hubs for young people (77). Encourage active participation in social activities and foster a sense of identity that contributes to the perception of happiness (78). Clearly, providing a variety of activity Spaces is more beneficial to young people seeking to engage in a variety of social and recreational activities.

On the other hand, the clean and comfortable environment of green space and the environment smell reduce negative emotions. According to psychological research, the senses have a great impact on the human brain’s acceptance of external information (79). Some studies have further indicated that young people’s concerns about smell are more obvious (80). According to our research, in green space, young people’s visual and olfactory perception has a more prominent impact on their emotions. Therefore, in the planning of green space, attention should be paid to visual and odor-related landscape construction to better improve the emotional health of young people.

The survey results also reflect some problems. At present, young people are reluctant to participate in green space activities, possibly because these activity Spaces are poorly designed and ignore key factors such as environmental cleanliness and smell. In order to effectively solve this problem, it is recommended to adopt strategies that meet the preferences of adolescents. This requires enhancing the diversity and comfort of green Spaces and optimizing the layout of greenery, including the introduction of challenging activity facilities for young people. By focusing on young people’s olfactory preferences and strategically planning plant configurations to attract their active participation.

### 4.2. Use of behavioral features

In conjunction with the analysis, it was found that as youths’ awareness of emotional health increased, so did the frequency of visiting green spaces, thereby improving youths’ emotional health gains and achieving a better emotional state. The study found that, in addition, young people were found to be more willing to improve their emotional health through social recreation. Fitness and game viewing in green space activities were found to be effective in increasing positive emotions and decreasing negative emotions among young people. Our findings are consistent with previous studies demonstrating the positive effects of physical activity on emotional regulation (81). To achieve this, green areas can be equipped with additional sports facilities, such as sports equipment and plastic running tracks, to better relieve the pressure on users.

In addition, passive activities such as viewing landscapes contribute to emotional well-being, possibly because young people use green Spaces as a space to escape social pressures and think (82). Previous studies have shown that waterscape viewing can improve young people’s mental health by providing them with a sense of safety and shelter (83, 84). Green space planners and designers can add some landscape pieces, viewing platforms, pro-level platforms and other ornamental spaces to improve the ornamental value of green space, which can effectively reduce negative emotions.

In China’s high-density cities, the use of public transportation and walking has been proved to reduce negative emotions, which may be partly due to the fact that public transportation is effective in alleviating traffic congestion and improving parking accessibility, thereby avoiding the time of traffic delay (85). On the other hand, walking has a positive effect on the social and physical health of residents (86). To encourage sustainable, low-carbon transport options, urban public green Spaces can benefit from increased installation of public transport facilities, further promoting environmentally friendly modes of travel.

### 4.3. Demographic characteristics

In terms of gender, Greenfield cognition has different impacts on emotional well-being. According to the previous Wilder’s Prime Law (87), women are more susceptible to stress, and we find that Greenfield cognition has a greater impact on women’s negative emotions. In addition, the study found that Greenfield cognition had a greater impact on men’s positive emotions, and that men and women experienced and used green Spaces differently. Compared with girls, men are more enthusiastic about jogging and socializing in public green Spaces (88), which may have a more significant impact on positive emotions.

In terms of age, we found that the impact of green space perceived benefit on emotion was different in different age groups, and the younger group was more likely to perceive the health benefit, so it was easy to generate positive emotion; However, young people in the older adult group had less perception of health benefits and were more affected by negative emotions. When it comes to income, there is also a correlation between emotional health and groups with different incomes. This may be related to their different living environments, stress levels and purpose of visit (89). The younger age group is usually in school or just starting to work, without the strong family life pressure and economic pressure of the older age group, and their purpose of visiting is simple relaxation or social entertainment, while the older age group mostly accompany their children or family members, and rarely visit for the simple purpose of relaxation, so their perception of health benefits is weaker, and the impact of negative emotions is greater (90).

To improve the effects of gender, age and income on emotional well-being, green spaces can be designed to meet the needs of different demographics through a variety of green space configurations. For example, for women, more ornamental landscaping can be installed to relieve stress. Educational activities about the benefits of green spaces can be increased to encourage more young people to visit green spaces and improve their emotional health. Landscape architects can design more diverse green spaces for young people of different socio-demographic profiles while conducting education about the benefits of green spaces to promote young people’s access to green spaces and improve emotional health.

### 4.4. Limitations and research for the future

Using the latest research findings in psychology, statistics, and ecology, as well as regression equations, structural equations, and multigroup analysis methods, this paper examines how the way young people view and use green space affects their emotional well-being from different perspectives. This is a meaningful attempt.

However, this study only examined one point in time, and from a cross-sectional perspective, it is impossible to distinguish the causal relationship. Most of the interviewed residents in this study were young urban residents, highly educated, and were more low-income people aged 18–25, so the sample characteristics were not comprehensive enough. Secondly, the sample size was not large enough. It should be increased in the follow-up study to improve the coverage of the main sample and avoid the excessive concentration of sample characteristics. Meanwhile, in the questionnaire setting, more consideration should be given to tourists’ needs, the analysis index of urban green space and emotional health should be increased, and physical instruments can be used to measure emotional health values so as to explore tourists’ perceptions and higher-level needs of urban green space in a more scientific and objective way.

## 5. Conclusion

Young people, as a significant group of park users, often have different needs with respect to the health benefits of urban green space. We explored the potential relationship between youths’ use behavior, perceived health benefits, and emotional well-being of green spaces through a questionnaire survey. Our research has found that (1) in terms of perceived green benefits, youth were more concerned about the comfort and cleanliness of the environment, the smell of vegetation, and the adequacy of facilities. (2) The diversity of activity spaces, the aroma of vegetation, and the planning and creation of a comfortable and clean environment can be beneficial in improving the emotional well-being of young people. (3) Young people are more inclined to use and experience green spaces through physical exercise and scenic observation, thereby further enhancing their emotional well-being. While, Social entertainment within green spaces is also a favored activity among young people, as it can strengthen communication and interaction with peers, ultimately promoting the improvement of their emotional well-being. (4) The perception of green spaces, the purpose of visiting them, and emotional well-being are indeed interrelated. Additionally, we have observed that different demographic groups exhibit distinct usage preferences. Gender, age, and income are closely linked to the impact of green spaces on the emotional well-being of young residents.

The young people reported that they rarely visit parks in their daily lives, which may indicate a lack of awareness of green spaces, a weak understanding of the potential impact on emotional well-being, or insufficient recognition of their restorative effects. Our findings may therefore raise concerns about the restoration of outdoor activities for young people. Young people’s perceptions and use of parks and changes in their moods may be dependent on their lifestyles and interests; policymakers at the local level should consider organizing nature programs that align with youth’s orientations, strengthening the public’s awareness and education on the relevance of urban green spaces to young people, and considering their preferences for emotional well-being in green space design: maintaining environmental cleanliness and introducing the pleasant fragrance of plants to provide young people with a satisfying olfactory experience; ensuring an adequate number of sports and recreational facilities to meet the exercise needs of young people; diversifying the forms of activity spaces to satisfy the multifaceted curiosity of young individuals. Which could ultimately enhance youths’ perceptions of the environment and positive mood. Future research on green spaces for youth mental health could be expanded through the lens of emotional health, not only as a development of traditional mental health but also as a deeper understanding of the benefits of urban green spaces.

## Data availability statement

The original contributions presented in the study are included in the article/Supplementary material, further inquiries can be directed to the corresponding author.

## Ethics statement

Ethical review and approval was not required for the study on human participants in accordance with the local legislation and institutional requirements. Written informed consent from the participants was not required to participate in this study in accordance with the national legislation and the institutional requirements.

## Author contributions

HC: conceptualization, validation, writing–review and editing, supervision, and project administration. NL and JW: methodology and writing draft preparation. JW and JZ: software and visualization. YG: formal analysis. JW and NL: investigation. HC: resources. NL and JZ: data curation. All authors have read and agreed to the published version of the manuscript.
