# Supplementary material for: The health perception of urban green spaces and its emotional impact on young adults: an empirical study from three cities in China
Source: Front Public Health. 2023 Nov 2;11:1232216. doi: 10.3389/fpubh.2023.1232216 (PMC10651752; doi:10.3389/fpubh.2023.1232216)
Supplement: Supplementary file 1 [file Data_Sheet_1.docx]

Supplementary Material


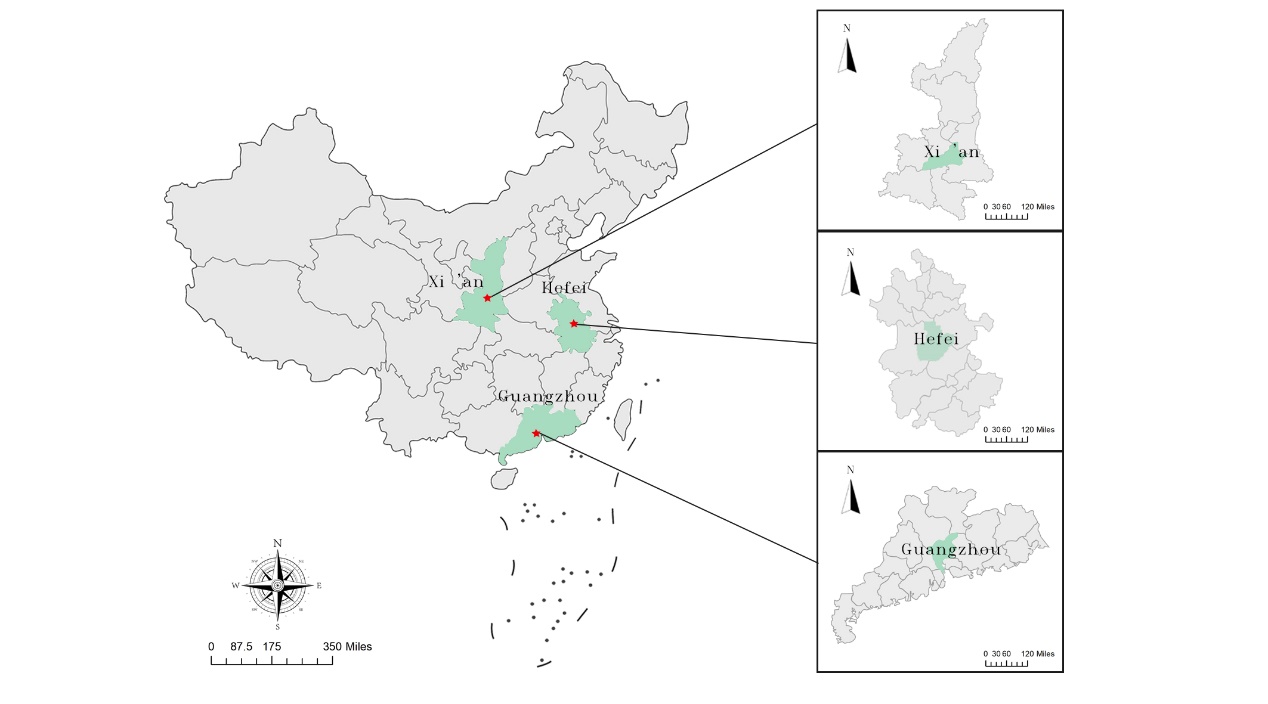


Figure 1. Location of the questionnaire.

Figure 2. Structural equation model diagram.

Table 1. Level of green space in the three cities

| City | Green area  (m^2^ /person) | | Green cover  (%) | | Green area  (hectares). | | year-end population  (million) | |
| --- | --- | --- | --- | --- | --- | --- | --- | --- |
| Hefei | | 10.41 | | 44.18 | | 25195 | | 946.50 |
| Guang zhou | | 17.20 | | 38.26 | | 59558 | | 1881.06 |
| Xi'an | | 11.80 | | 42.72 | | 46466 | | 1316.30 |

Note: Green coverage and green area indicate built-up areas.

Table 2. Independent Variables

| **Variable** | **Category** | **Description** | **Indicator reference** |
| --- | --- | --- | --- |
| **Spatial properties** | Green space activity spaces | activity plazas and open spaces | [55] |
|  | Pathway systems | Road planning, road paving | [55] |
| **Landscape attributes** | Botanical landscape | Color scheme of plants, number of species, ornamentation | [56] |
|  | Environmental smells | Plant odors and soil aromas | [57] |
|  | Environmental sounds | Animal calls, water flow, music | [58-59] |
|  | Environmental attractiveness | Environmental tidiness | [60] |
|  | Animal groups | Wildlife in green spaces | [61] |
| **Facilities** | Management system | Environmental safety, activity arrangements | [62] |
|  | Resting facilities | Quality and quantity of chairs and seats | [55] |
|  | Recreational facilities | Quality and quantity of running tracks, sports facilities, interactive facilities | [63] |

Table 3. Demographic characteristics of participants.

| **Factor** | **Number** | **Ratio%** | **Factor** | **Number** | **Ratio%** |
| --- | --- | --- | --- | --- | --- |
| **Gender** |  |  | **Way of travel** |  |  |
| Male | 158 | 37% | Walking | 182 | 42.7 |
| Female | 268 | 63% | Bicycles | 90 | 21.1 |
| **Age** |  |  | Electric vehicles | 43 | 10.1 |
| 18-25 | 254 | 60% | Cars | 21 | 4.9 |
| 26-30 | 158 | 37% | Motorcycles | 4 | 0.9 |
| 31-35 | 14 | 3% | Public transportation and subway | 86 | 20.2 |
| **Career status** |  |  | **Time consumption** |  |  |
| Waiting for employment | 19 | 5% | 1 to 15 minutes | 168 | 39.4 |
| Current students | 360 | 85% | 16 to 30 minutes | 164 | 38.5 |
| On-the-job | 47 | 11% | 31 minutes - 1 hour | 59 | 13.8 |
| **City** |  |  | More than one hour | 35 | 8.2 |
| Hefei | 154 | 36% | **Visit frequency** |  |  |
| Guangzhou | 141 | 33% | Once or twice a week | 389 | 91.3 |
| Xi'an | 131 | 31% | Three to five times a week | 26 | 6.1 |
| **Monthly income (RMB)** |  |  | More than five times a week | 11 | 2.6 |
| <1,000 | 133 | 31% | **Event length** |  |  |
| 1,000-2,000 | 192 | 45% | Morning: 5:00-8:00am | 23 | 5.4 |
| 2,000-3,000 | 13 | 3% | Morning: 8:00am-11:00am | 38 | 8.9 |
| 3,000-5,000 | 33 | 8% | Noon: 11:00-13:00 | 13 | 3.1 |
| 5000-10,000 | 29 | 7% | Afternoon: 13:00 - 16:00 | 48 | 11.3 |
| >10,000 | 26 | 6% | Evening: 16:00-19:00 | 174 | 40.8 |
| **Activity type** |  |  | Evening: 19:00-24:00 | 130 | 30.5 |
| Walking | 375 | 31.10% | **Access time** |  |  |
| Rest | 251 | 20.80% | Less than half an hour | 117 | 27.5 |
| Fitness | 139 | 11.50% | Half an hour to one hour | 201 | 47.2 |
| Social | 190 | 15.80% | One hour - two hours | 82 | 19.2 |
| View | 250 | 20.70% | Two hours - four hours | 21 | 4.9 |
|  |  |  | More than four hours | 5 | 1.2 |

|  | Average value | Standard deviation | Positive emotions | Negative emotions | Event space | Road system | Attractiveness of the environment | Environmental odor | Environmental voices | Management system | Plant landscape | Animals | Number of facilities | Facility quality |
| --- | --- | --- | --- | --- | --- | --- | --- | --- | --- | --- | --- | --- | --- | --- |
| Positive emotions | 15.876 | 3.71 | 1 |  |  |  |  |  |  |  |  |  |  |  |
| Negative emotions | 9.683 | 5.005 | 0.143** | 1 |  |  |  |  |  |  |  |  |  |  |
| Event space | 3.448 | 1.145 | 0.142** | 0.023 | 1 |  |  |  |  |  |  |  |  |  |
| Road system | 3.42 | 1.114 | 0.108* | 0.02 | 0.711** | 1 |  |  |  |  |  |  |  |  |
| Attractiveness of the environment | 3.847 | 1.057 | 0.031 | -0.191** | 0.614** | 0.576** | 1 |  |  |  |  |  |  |  |
| Environmental odor | 3.8 | 1.131 | 0.041 | -0.200** | 0.578** | 0.593** | 0.765** | 1 |  |  |  |  |  |  |
| Environmental voices | 3.589 | 1.084 | 0.091 | -0.051 | 0.534** | 0.539** | 0.594** | 0.662** | 1 |  |  |  |  |  |
| Management system | 3.674 | 1.022 | 0.084 | -0.073 | 0.562** | 0.623** | 0.633** | 0.662** | 0.650** | 1 |  |  |  |  |
| Plant landscape | 3.66 | 1.06 | 0.106* | -0.113* | 0.500** | 0.528** | 0.661** | 0.665** | 0.701** | 0.636** | 1 |  |  |  |
| Animals | 3.406 | 1.072 | 0.115* | -0.001 | 0.432** | 0.469** | 0.439** | 0.548** | 0.608** | 0.577** | 0.658** | 1 |  |  |
| Number of facilities | 3.69 | 1.053 | 0.087 | -0.08 | 0.523** | 0.562** | 0.592** | 0.614** | 0.626** | 0.684** | 0.582** | 0.583** | 1 |  |
| Facility quality | 3.667 | 1.107 | 0.06 | -0.114* | 0.462** | 0.501** | 0.578** | 0.616** | 0.578** | 0.636** | 0.617** | 0.564** | 0.707** | 1 |
| Note: N=426,* P<0.05 ** P<0.01. | | | | | | | | | | | | | | |

Table 4. Correlation analysis between variables

Table 5. Analysis of elements that influence the use of green space features by young people.

| Variables | Select | Access frequency | Event length | Transportation | Time consumption |
| --- | --- | --- | --- | --- | --- |
| Demographics  features | Gender | -0.127*** | 1.848 | -0.945 | -0.086 |
|  | Age | 0.064 | -0.015 | 0.089 | 0.173 |
|  | Career | -0.280*** | 0.309** | 0.463* | -0.108 |
|  | City | 0.058* | -0.15 | 0.108 | 0.152 |
|  | Revenue | -0.023 | 0.187** | 0.482** | -0.041 |
| Purpose  of visits | Relieves stress and soothes the emotions | -0.018 | -0.119* | -0.082 | -0.039 |
|  | Exercise | 0.021 | 0.047 | 0.046 | 0.164* |
|  | Participation in recreational activities | -0.01 | -0.035 | 0.189 | -0.08 |
| Perceived benefits | Event space | -0.011 | 0.029 | 0.154 | -0.003 |
|  | Road system | -0.026 | -0.063 | 0.361** | -0.047 |
|  | Attractiveness of the environment | -0.01 | 0.125 | 0.33* | 0.09 |
|  | Environmental odor | 0.051 | -0.045 | -0.251 | -0.072 |
|  | Environmental sounds | 0.003 | 0.003 | 0.138 | 0 |
|  | Management system | 0.041 | 0.028 | -0.099 | -0.03 |
|  | Plant landscape | -0.055 | 0.003 | 0.152 | 0 |
|  | Animal groups | -0.005 | 0.05 | 0.09 | 0.095 |
|  | Number of facilities | 0.011 | -0.04 | 0.165 | -0.009 |
|  | Facility quality | -0.046 | -0.048 | -0.166 | -1.53 |

Note: ***, **, and * represent 1%, 5%, and 10% significance levels, respectively.

Table 6. Activity type multiple response frequencies.

| Activity type | Number  of  responders | Response percentage | Average  of  positive emotions | Average  of  negative emotions | χ² | P | df |
| --- | --- | --- | --- | --- | --- | --- | --- |
| Walking | 375 | 31.10% | 15.84 | 9.12 | 58.742 | 0.000** | 4 |
| Rest | 251 | 20.80% | 16.02 | 9.45 |  |  |  |
| Fitness | 139 | 11.50% | 16.18 | 10.07 |  |  |  |
| Social | 190 | 15.80% | 16.16 | 9.14 |  |  |  |
| View | 250 | 20.70% | 15.96 | 8.33 |  |  |  |

Note: ***, **, and * represent 1%, 5%, and 10% significance levels, respectively.

Table 7. Access frequency, activity time, and access time K-W H tests.

| Variables | Select | Positive emotions | | | Negative emotions | | |
| --- | --- | --- | --- | --- | --- | --- | --- |
|  |  | Rank average | H-test | Importance | Rank average | H-test | Importance |
| Access frequency | Once or twice a week | 209.63 | 5.27 | 0.072 | 209.1 | 6.478 | 0.039 |
|  | Three to five times a week | 265.87 |  |  | 250.04 |  |  |
|  | More than five times a week | 226.41 |  |  | 282.77 |  |  |
| Event length | Morning: 5:00-8:00am | 201.02 | 8.264 | 0.072 | 268.8 | 27.575 | 0.000** |
|  | Morning: 8:00am-11:00am | 234.36 |  |  | 241.08 |  |  |
|  | Noon: 11:00-13:00 | 210 |  |  | 317.62 |  |  |
|  | Afternoon: 13:00 - 16:00 | 236.74 |  |  | 247.33 |  |  |
|  | Evening: 16:00-19:00 | 221.38 |  |  | 202.56 |  |  |
|  | Evening: 19:00-24:00 | 190.83 |  |  | 187.4 |  |  |
| Access time | Less than half an hour | 209.74 | 0.356 | 0.986 | 236.54 | 14.654 | 0.005 |
|  | Half an hour to one hour | 214.13 |  |  | 199.01 |  |  |
|  | One hour - two hours | 217.79 |  |  | 198.67 |  |  |
|  | Two hours - four hours | 216.33 |  |  | 257.43 |  |  |
|  | More than four hours | 193.9 |  |  | 315.6 |  |  |

Note: ***, **, and * represent 1%, 5%, and 10% significance levels, respectively.

Table 8. Analysis of between-group differences in mode of transportation and time consumption.

| Variables | Select | Positive emotions | | | Negative emotions | | |
| --- | --- | --- | --- | --- | --- | --- | --- |
|  |  |  | Anova | |  | Wales | |
|  |  | M | F | P | M | F | P |
| Transportation | Walking | 15.67 | 3.49 | 0.054 | 9.40 | 4.192 | 0.006 |
|  | Bicycles | 15.9 | 3.66 |  | 9.68 |  |  |
|  | Electric vehicles | 14.62 | 3.49 |  | 9.79 |  |  |
|  | Cars | 16.66 | 5.04 |  | 12.52 |  |  |
|  | Motorcycles | 17.75 | 3.40 |  | 17 |  |  |
|  | Public transportation and subway | 16.61 | 3.80 |  | 9.18 |  |  |
| Time consumption | 1 to 15 minutes | 15.98 | 3.81 | 0.953 | 9.59 | 1.225 | 0.034 |
|  | 16 to 30 minutes | 15.75 | 3.57 |  | 9.75 |  |  |
|  | 31 minutes - 1 hour | 15.88 | 2.95 |  | 8.93 |  |  |
|  | More than one hour | 15.94 | 4.89 |  | 11.05 |  |  |

Table 9. Cronbach's alpha coefficients of latent variables for each dimension.

| latent variable | Alpha coefficient | latent variable | Alpha coefficient |
| --- | --- | --- | --- |
| Greenland awareness | 0.936 | Purpose of the visit | 0.741 |
| Positive emotions | 0.781 | Negative emotions | 0.934 |
| Overall | | 0.835 | |

Table 10. Model coefficients and significance estimation results.

| Factors (latent variables) | → | Analysis term (dominant variable) | Non-standardized coefficients (Coef.) | Standardization factor (standard estimate). | Standard error (Std. error) | Z | P |
| --- | --- | --- | --- | --- | --- | --- | --- |
| Greenland awareness | → | Positive emotions | 0.078 | 0.141 | 0.032 | 2.463 | 0.014** |
| Purpose of the visit | → | Positive emotions | -0.127 | 0.206 | 0.062 | -2.027 | 0.043* |
| Greenland awareness | → | Negative emotions | 0.195 | -0.106 | 0.061 | 3.181 | 0.001*** |
| Purpose of the visit | → | Negative emotions | 0.380 | 0.186 | 0.120 | 3.177 | 0.001*** |
| Note: ***, **, and * represent 1%, 5%, and 10% significance levels, respectively. | | | | | | | |

Table 11. Results of multi-group analysis of youth demographic characteristics.

| Measurement path | Demographic characteristics of young residents | | | | | | |
| --- | --- | --- | --- | --- | --- | --- | --- |
|  | Male | | Women | Underage | advanced age | low income | High income |
| Positive emotions <-- Greenfield perception | | 0.121* | 0.060 | 0.075 | 0.053 | 0.075 | 0.053 |
| Negative emotions <-- Greenfield's view | | -0.152 | -0.097 | -0.081 | -0.194* | -0.081 | -0.194* |
| Positive emotions <-- Purpose of the visit | | 0.240 | 0.129 | 0.268** | 0.102 | 0.268** | 0.102 |
| Negative emotions <-- Purpose of the visit | | 0.689 | 0.181 | 0.326* | 0.475* | 0.326 | 0.475* |
| χ²/df | | 2.567 | | 2.193 | | 2.000 | |
| Liaoning university | | 0.924 | | 0.913 | | 0.913 | |
| NFI | | 0.861 | | 0.853 | | 0.853 | |
| IFI | | 0.924 | | 0.901 | | 0.914 | |
| RMSEA | | 0.061 | | 0.053 | | 0.053 | |

Note: ***, **, and * represent 1%, 5%, and 10% significance levels, respectively.

Table 12. Results of multi-group analysis of the frequency and activity time of young people visiting green spaces.

| Measurement path | Access frequency | | Event time | |
| --- | --- | --- | --- | --- |
|  | Low Frequency | High frequency | Low duration | High duration |
| Positive emotions <-------------------- Greenfield's view | 0.126 | -0.201 | 0.082* | 0.07 |
| Negative emotions <-------------------- Greenfield's view | -0.192 | 0.577 | -0.168* | -0.021 |
| Positive emotions <-------------------- Purpose of the visit | 0.176 | 0.446 | 0.198** | 0.296 |
| Negative emotions <-------------------- Purpose of the visit | 0.304 | 0.831 | 0.265* | 0.901* |
| x^2^ /df | 2.256 | | 1.87 | |
| Liaoning University | 0.915 | | 0.935 | |
| NFI | 0.854 | | 0.872 | |
| IFI | 0.916 | | 0.936 | |
| RMSEA | 0.052 | | 0.045 | |

Note: ***, **, and * represent 1%, 5%, and 10% significance levels, respectively.
